# Yeast Genome Maintenance by the Multifunctional PIF1 DNA Helicase Family

**DOI:** 10.3390/genes11020224

**Published:** 2020-02-20

**Authors:** Julius Muellner, Kristina H. Schmidt

**Affiliations:** 1 Department of Cell Biology, Microbiology and Molecular Biology, University of South Florida, Tampa, FL 33620, USA; jmuellner@mail.usf.edu; 2 Cancer Biology and Evolution Program, H. Lee Moffitt Cancer Center and Research Institute, Tampa, FL 33612, USA

**Keywords:** Rrm3, Pif1, Pfh1, G4 structures, DNA replication, rDNA, replication fork pausing, genome instability

## Abstract

The two PIF1 family helicases in *Saccharomyces cerevisiae,* Rrm3, and ScPif1, associate with thousands of sites throughout the genome where they perform overlapping and distinct roles in telomere length maintenance, replication through non-histone proteins and G4 structures, lagging strand replication, replication fork convergence, the repair of DNA double-strand break ends, and transposable element mobility. ScPif1 and its fission yeast homolog Pfh1 also localize to mitochondria where they protect mitochondrial genome integrity. In addition to yeast serving as a model system for the rapid functional evaluation of human Pif1 variants, yeast cells lacking Rrm3 have proven useful for elucidating the cellular response to replication fork pausing at endogenous sites. Here, we review the increasingly important cellular functions of the yeast PIF1 helicases in maintaining genome integrity, and highlight recent advances in our understanding of their roles in facilitating fork progression through replisome barriers, their functional interactions with DNA repair, and replication stress response pathways.

## 1. History of the PIF1 DNA Helicase Family 

**Figure 1:**
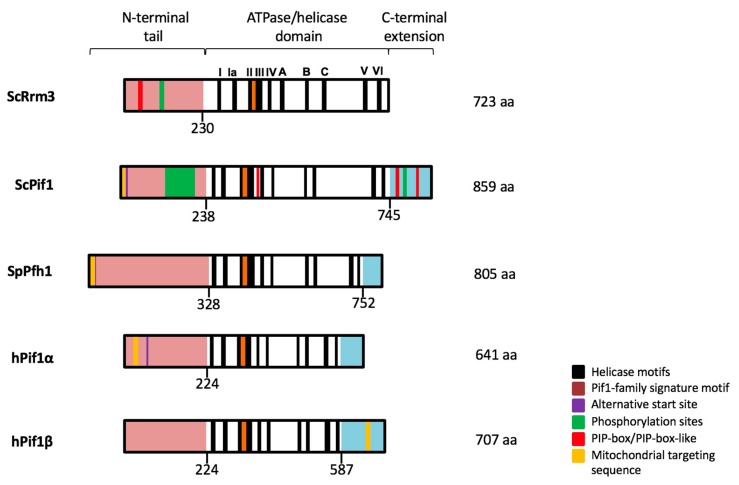
Structure and functional motifs of the yeast and human PIF1 family helicases. *Saccharomyces cerevisiae* expresses two members of the PIF1 family, Rrm3, and ScPif1, whereas *Schizosaccharomyces pombe* and higher eukaryotes express one. PIF1 helicases share the conserved ATPase/helicase domain and an intrinsically disordered N-terminal tail of variable sequence. Post-translational modification sites, proliferating cell nuclear antigen (PCNA)-interacting protein (PIP) box and alternative start sites, which give rise to mitochondrial isoforms, are marked [9,10,11,12,13,14].

The PIF1 DNA helicase family is conserved from bacteria to mammals [1,2]. While the yeast *Schizosaccharomyces pombe* and more complex multicellular eukaryotes, including humans, only encode one PIF1 family helicase, *Saccharomyces cerevisiae* expresses two: Rrm3 and ScPif1. The *ScPIF1* gene was originally identified in a screen to determine mutations that change the recombination frequency of tandemly arrayed repeats within mitochondria, and was therefore named after that defect, *p*etite *i*ntegration *f*requency (Sc*PIF1*) [3]. In a quest to determine genes that suppress recombination of tandem repeats of the ribosomal DNA (rDNA) and copper chelatin (*CUP1*) genes in *S. cerevisiae*, the *r*ibosomal DNA *r*ecombination *m*utation 3 (*RRM3*) gene was identified and later classified as a member of the PIF1 DNA helicase family based on sequence similarity [4,5]. Both Rrm3 and ScPif1 belong to the superfamily 1B and have 5′–3′ translocase activity that is encoded in helicase domains that share 40% identical residues [4,6,7,8]. An overview of the domain structure and functional motifs of PIF1 helicase family members in yeasts and humans is provided in Figure 1. This leaves the intrinsically disordered N-terminal extensions of Rrm3 and ScPif1 to regulate their enzymatic activity and their recruitment to specific sites within the yeast genome, where they perform the many distinct cellular functions discussed in this review. 

## 2. Replication Through the rDNA Replication Fork Barrier

After initiation of replication of the highly repetitive ribosomal DNA (rDNA) locus that spans approximately 1.5 Mb on chromosome XII, the leftward-moving replication fork encounters a cis-acting sequence near the 3’ end, called the replication fork barrier (RFB). RFB is located in a non-transcribed spacer and contains two termination sites, Ter1 and Ter2, that are bound by Fob1 to ensure that replication of the rDNA locus occurs in a unidirectional manner [15]. The PIF1 helicase family members in yeasts whose functions are fairly well-understood (Rrm3, ScPif1, SpPfh1) all accumulate at the RFB and 35S regions of the rDNA, indicating an intimate involvement in the regulation of rDNA replication [16,17]. However, they appear to have opposite effects; Rrm3 and Pfh1 promote replication through the RFB whereas ScPif1 maintains it, although the molecular mechanisms by which they modulate the RFB remain unclear [16,17]. These distinct roles in rDNA replication are evidenced by the increased number of chromosomal rDNA repeats, converged forks and fork pausing in *rrm3* and *pfh1* mutants when compared to *pif1* mutants or wildtype cells [16,18]. It is thought that Rrm3’s helicase activity removes DNA-bound proteins, including Fob1, ahead of the replication forks to prevent pausing [18,19]. However, deletion of *FOB1* only partially restores replication fork movement through the rDNA locus in *rrm3* mutants, indicating the presence of Fob1-independent barriers, such as the 35S and 5S rRNA genes and the inactive ARS [18,20]. Additionally, removal of RFBs by deletion of *FOB1* cannot rescue lethal interactions of *rrm3* with deletions of the RecQ helicase gene *SGS1*, or the fork protection complex gene *MRC1*, indicating that forks stalled at RFB and their intermediates are not toxic to *sgs1*Δ and *mrc1*Δ mutants, and that Rrm3 performs crucial functions at genomic loci besides RFB [18,20]. Two other subunits of the fork protection complex, Tof1 and Csm3, actually inhibit the “sweepase” activity of Rrm3 to remove DNA-bound proteins at termination sites of chromosomal rDNA and RNA polymerase III transcription [20], either directly by inhibiting the helicase activity of Rrm3 or indirectly by causing a conformational change of the replisome leading to restriction of Rrm3 activity [20]. Although disruption of the fork protection complex abolishes pausing at termination sites, further deletion of *RRM3* leads to a partial re-establishment of the termination site similar to the observation in *rrm3 fob1* mutants [18,20]. This is consistent with the observation that replication forks in *rrm3* mutants also stall at Fob1-independent sites. Other DNA helicases, such as Sgs1 and Srs2, which are also capable of removing DNA-bound proteins [21,22], were dispensable for replication through RFB [20]. This raises the possibility that the factors that promote fork escape in *rrm3 tof1* and *rrm3 csm3* mutants may not be another DNA helicase, but could involve DNA motor proteins that remodel DNA or chromatin.

**Figure 2:**
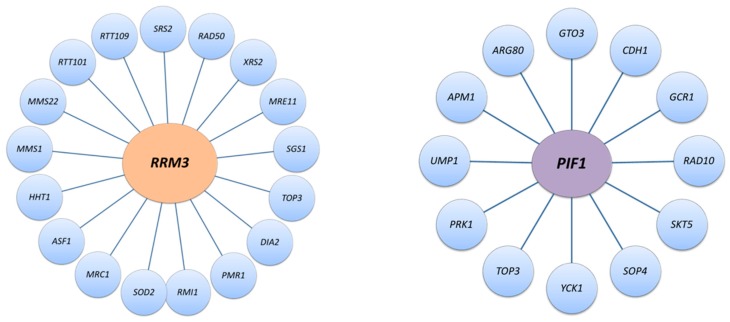
Synthetic lethal interactions of *RRM3* and *ScPIF1* [25,26,34,49,58,59,60,61,62,63,64,65,66,67,68,69,70,71,72,73,74,75,76,77,78,79].

**Figure 3:**
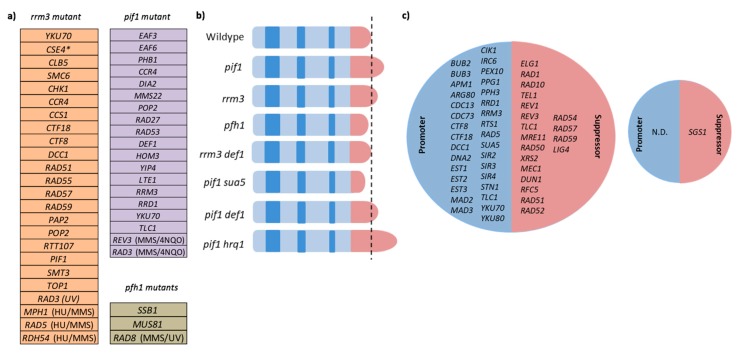
Functional interactions of *RRM3*, Sc*PIF1*, and *PFH1*. (**a**) Synthetic growth defects of *rrm3*, *pif1,* and pfh1 (pfh1-mt* and *pfh1-R20*) mutants. *Overexpression of *CSE4* leads to a growth defect in the *rrm3* mutant. [51,54,55,62,69,75,77,80,81,82,83,84,85,86,87,88,89,90,91,92]. (**b**) Effects of *pif1*, *rrm3,* and *pfh1-m21* mutations on telomere length [93,94,95,96]. Telomeres are depicted in pink and genes are in blue stripes (**c**) Genes that promote or suppress gross-chromosomal rearrangement formation in *pif1* (left) and *rrm3* (right) mutants. [25,34,48,91,94,97,98,99,100,101,102,103,104,105].

**Figure 4:**
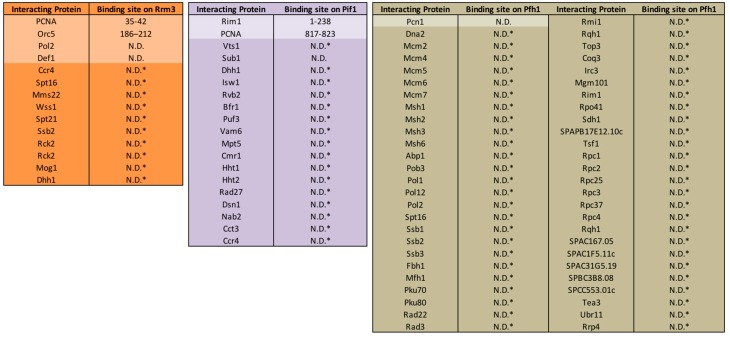
Physical interactions of Rrm3, ScPif1, and Pfh1. Interactions of Rrm3, ScPif1, and Pfh1 with proteins in lighter colored boxes have been verified by yeast-two-hybrid and/or co-immunoprecipitation. Binding sites on Rrm3, ScPif1, and Pfh1 and the method of detection are indicated. Putative binding proteins shown in darker colored boxes were identified in various high-throughput affinity capture experiments [9,11,19,40,42,52,93,106,107,108,109,110,111,112,113,114,115,116,117,118,119,120,121,122,123,124,125,126,127,128,129,130].

Notably, unlike hydroxyurea (HU)-induced fork pausing, pausing at RFB is independent of the DNA-damage checkpoint kinases Mec1 and Rad53 and DNA synthesis resumes without breakage or recombination at RFBs [23], suggesting that naturally occurring replication pause sites are processed differently than those formed during DNA replication stress. This likely explains why deletion of *MRC1* does not affect replication fork pausing at Fob1-RFBs and why the *rrm3Δ mrc1-AQ* mutant, which is defective in the checkpoint function but not the replication function of Mrc1, is viable [23,24,25,26]. A better understanding of the chromatin environment in which natural barriers of DNA replication reside, compared to the environment established at genotoxin-induced paused forks will help to elucidate the mechanisms by which the mechanistically poorly understood Rrm3 and other PIF1 helicases contribute to genome maintenance and stability. An overview of the function of the yeast PIF1 helicases and their genetic and physical interactions is provided in Table 1 and Figure 2, Figure 3 and Figure 4.

## 3. Termination of DNA Replication

With the focus of the past decades on the role of the PIF1 helicase family in the elongation step of DNA replication, a role in DNA replication termination only emerged recently. Fork convergence and dissolution during replication termination is primarily the responsibility of the essential type II topoisomerase Top2. ScPif1 and Rrm3 are now recognized to contribute to fork convergence by unwinding the final stretch of parental DNA in a pathway independent of Top2 [53]. Consistent with this finding, Top2 was upregulated in the chromatin fraction of *rrm3*Δ cells [19]. Similarly in *S. pombe,* deletion of Pfh1 leads to short stretches of unreplicated DNA, which causes the accumulation of anaphase bridges, signaling problems with chromosome segregation [54,131]. 

## 4. Replication Through transfer RNA (tRNA) Genes 

In the absence of Rrm3, increased replication fork pausing is not only observed in the highly transcribed rDNA locus, but also in tRNA genes [18,20,34,35,38,132]. Pausing at tRNA genes is even more severe in the absence of ScPif1 [35,36], indicating that, in contrast to being dispensable for negotiating RFB, ScPif1 facilitates tRNA gene replication. However, extensive pausing of stalled replication forks at tRNA genes in *rrm3* mutants, but not in *pif1* mutants, and increased binding of ScPif1 to tRNA genes in the absence of Rrm3 suggest that ScPif1 plays mostly a backup role [35,36]. 

Since transcription continues during genome duplication in S-phase, Rrm3 and ScPif1 may also promote DNA replication by unwinding RNA-DNA hybrids that may occasionally form at highly transcribed genes. Indeed, ScPif1 and Rrm3 accumulate at higher rates at tRNA genes in the absence of ribonuclease H1 (Rnh1) when RNA-DNA hybrids, such as R-loops, accumulate; intriguingly, ScPif1 and Rrm3 possess higher affinity for DNA:RNA substrates than for duplex DNA [35,133]. In another model, head-on collisions between replisome and transcription machinery, rather than R-loops, are responsible for fork arrest in tRNA genes in the absence of Rrm3 and ScPif1 [34,36,134]. Replication forks also move more slowly through highly expressed RNAPII-transcribed genes. However, since pausing in these genes was not different in Rrm3-proficient or Rrm3-deficient cells as measured by Pol2 occupancy, increased Rrm3 association with these genes is most likely the result of its association with the replisome, which is enriched at these slower-replicating sites, rather than a direct role of Rrm3 in facilitating fork progression [38]. A subsequent study did suggest a role for Rrm3 at RNAPII-transcribed genes. In cells disrupted for the Facilitates Chromatin Transcription (FACT) complex, Rrm3 binding to longer and highly expressed RNAPII-transcribed genes increased, especially at the 3’end of open reading frames where R-loops are more likely due to increased fork impediments, suggesting that Rrm3 is directly involved in resolving R-loops in a common pathway with the FACT complex [135]. 

Similar to Rrm3, Pfh1 binds to rDNA and tRNA genes, the RFB of rDNA, and mating type loci in *S. pombe* [17], suggesting that Pfh1 also facilitates fork progression through those sites. Indeed, in the absence of Pfh1, fork pausing at tRNA genes increased significantly, especially in those tRNA genes where replication and transcription are at risk of meeting head-on, or where converged forks accumulate [17]. Unlike Rrm3, however, Pfh1 not only associates with highly expressed RNAPII-transcribed genes, but also facilitates their replication [17]. The proposal that Pfh1, like Rrm3, travels with the replication fork is supported by physical interactions of Pfh1 with several replisome components [40]. 

## 5. Telomere Length Maintenance

ScPif1 was identified in a screen for mutations that inhibit *de novo* telomere formation and telomere elongation [30]. Indeed, ScPif1 associates with telomeres *in vivo*, and inhibits telomerase by removing it through its helicase activity, leading to the characteristically elongated telomeres of *pif1* mutants [13,31]. ScPif1 removes telomerase by interacting directly with the finger domain of the telomerase subunit Est2 [136]. Recently, ScPif1 was found to regulate telomere elongation by affecting the spatial distribution of telomerase components by segregating the limiting component of telomerase, the RNA TLC1, to the nucleolus [44]. While it is known how ScPif1 removes telomerase from telomere DNA, the mechanism of sequestering it to the nucleolus remains to be identified. While *rrm3* mutants also have slightly elongated telomeres, they do not appear to be the result of increased telomerase activity, but most likely stem from replication problems [27]. Notably, Rrm3 physically interacts with Def1, a protein of unknown biochemical activity that is required for normal telomere length maintenance [93]. However, the functional significance of this interaction for the replication of telomeres or other genomic sites is unknown. The effect of *pif1*, *rrm3,* and *pfh1* mutations on telomere length is summarized in Figure 3b. 

During telomeric replication, ScPif1 generates single-stranded DNA (ssDNA) of the 3’ end by regulating a nuclease activity that functions in parallel to the 5’–3’ exonuclease Exo1 [75]. This nuclease remains to be identified, but a potential candidate is Dna2, which is also needed for Okazaki fragment maturation, and acts with the Sgs1 helicase in the long-range resection of DNA double-strand breaks to produce 3’ overhangs for homologous recombination [75,137]. The possibility that ScPif1 possesses nuclease activity was recently tested as it contains the conserved residues for 3′–5′ exonuclease activity in its helicase core domain; however, cleavage was negligible even at extremely high concentrations [138]. These residues are not conserved in Rrm3 or hPif1, indicating that other members of the PIF1 family are also unlikely to possess exonuclease activity. 

Following senescence, two types of survivors with altered telomere structures can be identified in yeast cells. Type I survivors have short telomeric TG_1-3_ tracts with highly amplified subtelomeric Y-elements, and Type II survivors have long heterogeneous telomeric TG_1-3_ tracts, which are generated by homologous recombination (HR) and by break-induced replication (BIR), respectively [75,139,140]. Interestingly, *pif1* mutants emerging from senescence do not clearly fall into either category, suggesting that ScPif1 is involved in both survival pathways [75]. Another study showed a stronger requirement of ScPif1 for the formation of recombinant-dependent Type I survivors [141]. 

Just recently, Hrq1, a DNA helicase that has similarity to the human RecQ helicase RecQL4 [142], was shown to functionally interact with ScPif1 to synergistically modulate telomere length homeostasis by regulating telomerase activity [143]. Depending on whether the ScPif1 helicase or the Hrq1 helicase was active, telomerase was either inhibited or stimulated, and the authors propose that post-translational modifications that modulate the helicase activity of either one, such as already shown for ScPif1 [144,145], could thus regulate telomerase activity [143]. 

Broken chromosomes can be repaired by homologous recombination and non-homologous end-joining, or be healed by *de novo* telomere addition [146]. While *de novo* telomere additions at DNA-double-strand breaks (DSBs) are rare, their frequency and distribution are increased in the absence of ScPif1, but not in the absence of Rrm3 [27,30,105,146]. The drastic increase of *de novo* telomere additions in the *pif1* mutant is significantly reduced by *RRM3* deletion, emphasizing their differing modes of action at telomeres and in DSB repair pathways [27]. 

Depending on where in the genome a DSB forms, long-rage resection can lead to the exposure of single-stranded DNA sequences that resemble G-rich telomere-like repeats [147]. Based on its mechanism at telomeres, ScPif1 most likely inhibits *de novo* telomere additions at DSBs by preventing telomerase from binding to these repeats. Indeed, ScPif1 localizes to DNA damage foci at DSBs, indicating a direct role in their DNA repair. The Mec1/Rad53 signaling pathway has been identified to regulate *de novo* telomere addition at DSBs, with phosphorylated ScPif1 ensuring telomerase removal and phosphorylated Cdc13 inhibiting its recruitment [91,144]. However, how ScPif1 is recruited to DSBs is unknown. In addition to phosphorylation specifically in response to DSBs, a basal level of ScPif1 phosphorylation is observed that is independent of the DNA damage signaling pathway [144], but its source and functional significance are unclear. 

ScPif1 overexpression causes accumulation of ssDNA and activates the Rad53-dependent DNA-damage checkpoint. Excessive removal of telomerase, such as by ScPif1, leads to C-strand degradation in an Exo1-dependent manner [78,148]. Thus, in addition to regulating activity by post-translational modification, expression levels of ScPif1 are tightly regulated, with low levels in G1/S phase and high levels in late S/G2 [78]. In contrast, overexpression of Pfh1 in *S. pombe* leads to longer telomeres and therefore is considered a positive regulator of telomere length and replication [32].

## 6. Okazaki Fragment Maturation

In *S. cerevisiae*, Okazaki fragments are initiated by DNA polymerase α/primase, which generates 10-nucleotide long RNA primers followed by 10-20 nucleotides of DNA [149]. DNA polymerase δ extends this substrate until it reaches the 5’ end of the previous Okazaki fragment. A flap is generated during the removal of the RNA primer, which is ultimately removed by the flap endonuclease Fen1/Rad27, leading to a gap that is resynthesized and sealed by DNA ligase I [150,151]. However, *in vitro* longer flaps of 20–30 nucleotides also form, which cannot be processed by Fen1/Rad27 [152]. These longer flaps are coated by replication protein A, which inhibits Fen1/Rad27 nuclease activity but promotes Dna2 nuclease activity, which shortens the flaps [153,154,155]. After shortening, Fen1/Rad27 processes the final step to generate a nick, which is sealed by DNA ligase I [156]. A role for ScPif1 in Okazaki maturation has been proposed*,* as disruption of ScPif1 nuclear function, but not *RRM3* deletion, suppresses defects caused by *dna2-1* and *dna2-2* mutations as well as negative genetic interactions of *DNA2*, but not those with genes that have roles in Okazaki fragment processing [48]. Based on the genetic interaction between *PIF1* and *DNA2*, Budd et al. [48] propose that ScPif1 stimulates flap formation and thereby enforces the two-nuclease pathway of Okazaki fragment maturation that requires Dna2 and Fen1/Rad27. Thus, ScPif1 functions as a backup in Okazaki fragment maturation to shorten flaps when they escape from Fen1/Rad27. Besides ScPif1, Pol32 can also generate the long flaps for processing by Dna2 [157]. 

ScPif1 is also able to process excessive flaps that have folded back on themselves and cannot be processed by endonucleases [158]. These fold-back flaps possess a 5’ end that can be captured by the 5’–3’ helicase ScPif1 and unwound, including the DNA:RNA hybrid at the downstream primer [158]. This ScPif1 function is further supported by biochemical analysis of ScPif1’s helicase activity, which unwinds duplex DNA as efficiently as ssRNA-DNA flaps and is stimulated if a 5’ ssDNA substrate of at least 10 nucleotides is available [49]. It is also conceivable that flaps contain more complex secondary DNA structures, such as G-quadruplexes (G4 structures), that can be resolved by ScPif1. *S. pombe* Pfh1 and Rrm3 have also been implicated in Okazaki fragment maturation. Pfh1 functionally interacts with several Okazaki fragment maturation factors and, like ScPif1, has been implicated in flap processing, whereas Rrm3 appears to promote Pol δ processivity [36,49]. 

## 7. Resolution of G4 Structures 

A decade ago, ScPif1 was first shown to actively resolve G4 structures and prevent genome instability at repeats capable of forming G4 structures [159]. Besides association with DSBs and highly transcribed genes, ScPif1 is found at approximately 25% of G4 structures within the yeast genome, probably due to its preference for unwinding the rarer antiparallel G4s [39,78,160,161]. Depending on their stability, ScPif1 can unwind G4 structures in an ATP-dependent or ATP-independent manner. These preferences of ScPif1 suggest that other DNA helicases, such as those of the RecQ family, typically unwind the other, more common types of G4 structures [161,162,163,164]. 

Sub1, a co-transcriptional activator and suppressor of genome instability caused by transcription-induced negative helical torsion, and Mms1, a ubiquitin ligase component, are found at G4 structures where they may affect ScPif1 activity [119,165]. In particular Mms1, which preferentially binds lagging strand G4 structures, was shown to enhance ScPif1 binding at some G4s [165]. ScPif1’s role in resolving G4 structures likely extends to the mitochondrial genome, which has greater density of G4-motifs than the nuclear genome [39,166,167].

*S. pombe* Pfh1 is also found at G4-motifs and its depletion causes fork pausing, DNA damage, phosphorylation of histone H2A, and genome rearrangements [41,47]. Interestingly, most of the bound G4-motifs were found on the transcribed strand of highly transcribed RNAPII-transcribed genes, which could act not only as a recruiting platform for Pfh1 to unwind G4 structures, but also to resolve RNA-DNA hybrids to maintain replication-transcription stability [47]. 

ScPif1 resolves G4 structures by translocating on ssDNA as a monomer in an ATP-dependent manner to unwind the G-quadruplex one strand at the time, then waits at the ss/ds DNA junction where it dimerizes, transforming the translocase into a potent DNA helicase capable of unwinding dsDNA [163,168,169,170,171]. Monomeric ScPif1 appears to “patrol” sequences at risk of forming secondary structures by remaining in their proximity to repeatedly resolve G4 structures and RNA:DNA hybrids [171]. Constant unwinding of G4 structures could prevent dimerization of ScPif1 and thereby, acting as a regulatory substrate, inhibit ScPif1’s helicase activity. 

Notably, ScPif1 also possesses an annealing activity, which might anneal unwound G4 structures with their complementary strand to restore dsDNA and, thereby, inhibit G4 structure re-formation [160]. Though unresolved G4 structures act as barriers to DNA replication and can cause genome instability, sequences capable of adopting G4 structures are enriched at human origins of replication, gene promoters, telomeres, as well as in rDNA and the mitochondrial genome, suggestive of a functional role. Could ScPif1 regulate the formation of such functional G4 structures, using its annealing activity? Such regulation of G4 formation and G4 resolution could regulate expression of highly transcribed genes to avoid collisions with replication or repair machineries, establish replication fork barriers as an alternative to those formed by proteins, or modulate telomerase activity at telomeres.

Dahan et al. [12] have shown that ScPif1 is essential for replication through G4 structures on the lagging strand where its processivity is increased by its interaction with PCNA via its canonical PCNA-interacting protein (PIP) box. This interaction acts independently of the ScPif1-PCNA interaction during break-induced replication (BIR), which enhances DNA polymerase δ strand displacement synthesis via a non-canonical PIP sequence [12,52]. Thus, during unperturbed replication ScPif1 mostly resolves G4 structures on the lagging strand to suppress DNA breakage and maintain replication fork progression [39]. Resolving or repairing G4 structures in *pif1* mutants is error-prone, leading to G4-motifs that can no longer form G-quadruplexes [39,172]. 

The recently published crystal structure of hPif1 shows a conserved ssDNA binding channel that is important for DNA unwinding, but not for G4-DNA binding or DNA annealing [45]. In fact, mutations within the ssDNA binding channel enhance annealing activity as they reduce helicase activity [45]. Although such mutagenesis studies are beginning to shed light on the relationship between the annealing and unwinding activities, the biological significance of the annealing activity of PIF1 family helicases, and that of other DNA helicases with this activity, such as the RecQ family helicases Sgs1 and BLM, remains enigmatic.

## 8. Cellular Response to Replication Fork Stalling in the Absence of Rrm3

Populations of *rrm3Δ* cells exhibit a cell cycle defect with a DNA content intermediate between 1N and 2N, indicative of problems with timely progression through S-phase [83]. Deletion of *SRS2* or *SGS1* enhances this S-phase progression defect and causes a severe fitness defect that can be rescued by disrupting HR genes *RAD51* or *RAD55*, suggesting a role for Rrm3 either in preventing the formation of replication-dependent HR substrates or contributing to their repair [62,83,173]. In contrast, the synthetic lethality between deletions of *RRM3* and *MRE11* or *RAD50,* which code for subunits of the Mre11-Rad50-Xrs2 (MRX) DSB repair complex, is not due to illegitimate HR since it could not be rescued by *RAD54* or *RAD55* deletions [83]. This suggests that, in addition to HR, Rrm3 functions in another MRX-mediated pathway, such as non-homologous end joining, telomere maintenance or S-phase checkpoint activation. 

How cells deal with HR substrates and other replication problems that arise from replication forks paused at thousands of protein-bound sites in Rrm3-deficient cells is poorly understood. Syed et al. [19] used Stable Isotope Labeling with Amino Acids in Cell Culture (SILAC), coupled to mass spectrometry, to determine that topoisomerase Top2 and the SWI/SNF ATPases Rad5 and Rdh54 were significantly upregulated in cells lacking Rrm3, implicating their role in replication stress tolerance. Increased Top2 most likely compensates for the loss of Rrm3’s contribution to resolving converging replication forks [53]. Rdh54 is important for HR in diploid cells, but roles in haploid cells are also emerging. Notably, the recently identified role for Rdh54 in regulating D-loop formation [174], and thereby HR levels, could become increasingly important in the absence of Rrm3 when greater numbers of HR substrates are likely to form at replication pause sites. That Sgs1-Top3-Rmi1 and Srs2 define two independent pathways of D-loop reversal could contribute to the synthetic lethality of the *rrm3*Δ mutation with *sgs1*Δ and *srs2*Δ mutations as well as its suppression by *RAD51* deletion [62,83,173,174]. Upregulation of Mph1, which also functions in D-loop disassembly [175], was also observed in the *rrm3*Δ mutant, albeit to a lesser extent than Top2, Rdh54 and Rad5, and further supports the increased requirement for tight regulation of D-loop formation in the absence of Rrm3 [19,175]. 

Rad5 has replication fork reversal activity [176,177] and its upregulation in the *rrm3*Δ mutant may indicate that fork reversal is a major mechanism to restart forks that are stalled at protein barriers [19]. Considering the association of Rrm3 with replisome components Polε and PCNA [9,106], one could also speculate that Rrm3 itself can facilitate fork reversal to allow the forks to pass through protein-bound sites in a pathway that functions in parallel to replication stress-induced Rad5-mediated fork reversal. 

In the absence of Rrm3, the Rad53-dependent DNA-damage checkpoint is activated in a Rad9-dependent manner and remains active even after preventing the formation of HR intermediates by deleting *RAD51* [62,83,178]. During replication stress, Rrm3 itself is phosphorylated in a Rad53-dependent manner; however, phosphorylation is not required for replication across natural pause sites [10]. The biological function of Rrm3 phosphorylation remains unknown, but has been suggested to inhibit Rrm3 activity to prevent genome instability during replication stress [10]. 

## 9. A Helicase-Independent Function of Rrm3 During Replication Stress

During replication stress, cells lacking Rrm3 continue to progress into S-phase. The ability of Rrm3 to restrict DNA synthesis depends on the integrity of the 230-amino-acid long disordered N-terminal tail of Rrm3, but not its ATPase/helicase activity [19]. Increased nucleotide levels are not sufficient for S-phase progression as helicase-dead *rrm3* mutants also have increased dNTP levels, but do not progress into S-phase in hydroxyurea [19]. Notably, Rrm3 interacts with the origin recognition complex (ORC) subunit Orc5 and the region of the Rrm3 N-terminus required for inhibiting DNA synthesis during replication stress is required for the Rrm3-Orc5 interaction [19,110]. Moreover, the N-terminal tail of Rrm3 is required for its association with origins of replication during replication stress, but not during the unperturbed cell cycle, raising the possibility that Rrm3 acts at replication origins to restrict DNA synthesis during replication stress. 

## 10. Protection of Mitochondrial DNA

The *ScPIF1* gene encodes two ScPif1 isoforms that are translated from two different in-frame start codons. Mutating the second start codon leads to expression of the longer isoform, ScPif1-m2, which contains a mitochondrial signal sequence and localizes to the mitochondria [13,30]. ScPif1 and its homologue in *S. pombe,* Pfh1, move with the replisome during mitochondrial (mt)DNA replication and require their catalytic activity for efficient mtDNA replication [33,179]. Replication fork blockages in mtDNA cannot be resolved by increased dNTPs, but rather require a direct involvement of ScPif1 in fork progression [179]. Thus, while Rrm3 acts as a “sweepase” during nuclear replication, ScPif1 might perform the same function on mitochondrial DNA. 

Notably, Sc*PIF1* deletion leads to thermosensitivity and loss of mtDNA, which led to the suggestion that lack of ScPif1 association with the whole mitochondria genome leaves the DNA “naked” and unprotected [179]. However, the underlying mechanism for the temperature sensitivity remains unknown. Cellular levels of ScPif1 drastically decrease in the absence of mtDNA, leading the authors to propose that either ScPif1 is degraded in the mitochondria or the Sc*PIF1* gene is strongly down-regulated by the mitochondrial retrograde signaling pathway [180,181]. Moreover, ScPif1 and Pfh1 appear to protect the mitochondrial genome from DSBs or facilitate their repair [33]. ScPif1 prevents mtDNA mutagenesis from oxidative damage in cooperation with Ntg1 in a manner that is independent of recombination [182,183]. However, it remains to be determined how ScPif1 prevents oxidative base damage. Finally, the main cause for the petite phenotype of *PIF1* deletion mutants remains ambiguous, but probably involves gross deletions and rearrangements of the mitochondrial genome.

While the importance of ScPif1 for mitochondrial health in *S. cerevisiae* is firmly established, a mitochondrial role of Rrm3 is debated. A putative mitochondrial localization sequence has been proposed in Rrm3, and Rrm3 was identified in a screen for mitochondrially localized proteins in yeast, but not individually verified [29]. *RRM3* deletion causes an increase in mutagenesis of mtDNA [184] and suppresses mtDNA instability in the *pif1*∆ mutant by elevating the nucleotide pool through Rad53 activation [184,185]. Thus, even though ScPif1 and Rrm3 impact mtDNA integrity, they do so through different mechanisms and to a greatly different extent. This distinction is further highlighted by the finding that Rrm3 can alleviate the nuclear defects of a *PFH1* deletion, but not its mitochondrial defects [28].

## 11. Localization to Centromeres 

Besides associating with tRNA genes, telomeres and origins of replication, Rrm3 and ScPif1 were recently identified at centromeres [37]. This association is cell cycle regulated, with Rrm3 locating at centromeres from early to mid-S-phase and ScPif1 in late S/G2 phase, indicative of different roles for each helicase at centromeres, and requires the helicase activity of Rrm3, but not that of ScPif1 [37]. Curiously, in the absence of either Rrm3 or ScPif1, the remaining helicase appears to compensate by adopting the binding profile of the other. Furthermore, Rrm3’s centromere association, but not ScPif1’s, was stronger in *tof1* mutants, indicating a competitive relationship of Rrm3 and Tof1 for centromere affiliation [37]. Thus, even though both helicases are found at centromeres where they facilitate replication, their actions differ, with ScPif1 playing a backup role in the absence of Rrm3 as was previously observed at tRNA genes [36]. 

## 12. DNA Break Repair

Potential roles for ScPif1 in DNA repair, replication and recombination were indicated by its localization to DNA damage foci and preference for binding 3’ss/dsDNA junctions, which are DNA intermediates generated during these processes [171,186]. ScPif1’s function in BIR, which cells use for the repair of one-ended DSBs that can arise at eroded telomeres or when a replication fork encounters a single-strand nick in the template [187], is indicated by the increase in half cross-over products in the absence of ScPif1 [52]. During BIR, ScPif1 is found at and downstream of the site of strand invasion, which led to the suggestion that ScPif1 is directly involved in D-loop migration [52,188]. Binding of ScPif1 to PCNA, which stimulates strand displacement DNA synthesis by DNA polymerase δ, also stimulates BIR [11]. Furthermore, ScPif1’s processivity is regulated by force on ssDNA [189]. During BIR, the movement of the D-loop generates a greater force on the ssDNA and thereby increases the unwinding activity of ScPif1 [189]. ScPif1’s role in BIR is regulated by Rad53-mediated phosphorylation [145]. Recruitment of ScPif1 to DNA damage sites is poorly understood, but seems to depend on its physical interaction with PCNA [55]. There, ScPif1 facilitates the formation of ssDNA behind the replication fork to initiate template switching [55]. 

Muñoz-Galván et al. [51] recently determined that Rrm3 contributes to the repair of replication-dependent DSBs. The repair of these DSBs by sister-chromatid-recombination was significantly reduced in Rrm3-deficient cells whereas repair of replication-independent DSBs, such as those induced by homothallic switching endonuclease HO, were not affected by Rrm3. In contrast to ScPif1, DSB repair by Rrm3 occurred by a sister chromatid exchange mechanism, not BIR [51,52]. Rrm3 also localizes to replication-dependent DSBs and, similar to ScPif1, this association could be facilitated by its PIP-box-mediated physical interaction with PCNA [9,51]. 

## 13. Regulation of Ty1 Transposition

Rrm3 was originally identified as a suppressor of Ty1 transposition [5,56]. Potential sources for increased frequency of retro-mobility are higher levels of transcription of Ty1 or enhanced complementary DNA (cDNA) synthesis. In *rrm3* mutants multimeric Ty1 cDNA arrays form, possibly due to fork stalling at transposition-related RNA:DNA hybrids that cannot be resolved in the absence of Rrm3 [57]. Indeed, RNAse H1 overexpression prevented Ty1 multimers. Increased replication stress caused by simultaneous depletion of Rrm3 and Tof1, a component of the fork protection complex, caused a dramatic increase in Ty1 transposition [190]. In addition to increased transposition, *rrm3Δ* cells show increased integration at tRNA genes, highlighting Rrm3’s role not only in the suppression of Ty1 mobility and multimerization, but also in integration site selection [190].

Despite its major role in suppressing Ty1 and rDNA rearrangements, deletion of *RRM3* does not have a major effect on the accumulation of spontaneous gross-chromosomal rearrangements (GCRs) [25,191]. Upon induction of replication stress by HU, however, the GCR rate of the *rrm3*Δ mutant increased 7-fold, indicative of a role at stalled or paused forks [19]. Rrm3 also strongly suppresses homologous-recombination-dependent GCRs in cells lacking the RecQ family helicase Sgs1, consistent with a recent report of a role of Rrm3 in DSB repair [25,51,191]. An overview of suppressors and promoters of GCRs in *pif1* and *rrm3* mutants is provided in Figure 3c.

## 14. Fragile Site Expression

Naturally occurring fragile sites, such as the replication slow zone (RSZ) in the yeast genome, can cause DSBs during unperturbed replication [192]. While deletion of *RRM3* leads to fragility as a result of replication fork stalling at certain non-nucleosomal DNA-bound proteins, such as Fob1, it suppressed the expression of RSZ [18,178]. Rrm3 also reduced chromosome breakage at RSZs in a *mec1* mutant by preventing DSB formation at RSZs [178]. In contrast to *rrm3Δ*-sensitive fragile sites that are caused by impeded replication forks encountering non-nucleosomal DNA-bound proteins, fragile site expression in RSZs is the result of depletion of the dNTP pool [178], suggesting that two types of fragile sites exist within the yeast genome: dNTP-sensitive (RSZ) and *rrm3*-sensitive (tRNA genes, RFB, rDNA, telomeres) [178]. 

## 15. Yeast as a Model System for the Functional Evaluation of hPif1 Mutations

Human Pif1 has been suggested to act as a tumor suppressor [193]. Multiple *PIF1* variants that code for single amino changes of uncertain significance have been identified in cancer patients and L319P functionally evaluated in yeast. The completely conserved L319 is located in the helicase domain of hPif1 and mutation of its corresponding residue in Pfh1 was lethal, suggesting that it disrupts both nuclear and mitochondrial functions of Pfh1 and that L319P likely inactivates hPif1 [193]. Other hPif1 mutations from cancer genomes map near conserved helicase motifs (S223T) or affect other relatively conserved residues in the helicase domain (P357L, R592C), suggesting that they could also impair hPif1 function [193]. P109S, although located far upstream of the conserved helicase domain, also affects a completely conserved residue, but of unknown function [193]. The physical interaction between hPif1 and Brca1 in the resolution of G4 structures also supports a potential role for hPif1 in cancer suppression [194].

## 16. Concluding Remarks

Even though we know that PIF1 DNA helicases associate with over a thousand discrete sites in the yeast genome, including 274 tRNA genes and ∼900 sites in the rDNA array, as well as replication origins, boundary elements, and sites of replication fork convergence, some of the underlying mechanisms by which helicases of the PIF1 family prevent fork stalling at these sites remain unknown [34,35,36,38,195]. Despite Rrm3’s and ScPif1’s common binding regions in the yeast genome, such as tRNA genes, rDNA locus, centromeres, and telomeres, their mode of action at those sites appears distinct. Their highly disordered N-terminal tails, which are not conserved at the amino acid level, may be responsible for this difference by recruiting distinct sets of genome maintenance factors and being subject to distinct post-translational modification. Indeed, the recent identification of Rrm3 functions that map to the N-terminal tail rather than the helicase domain, N-terminal phosphorylation sites that regulate the helicase activity, and distinct as well as shared N-terminal binding partners keep adding to the ever-expanding properties of the PIF1 helicase family and their roles in maintaining genome integrity in unperturbed and stressed cells [10,19,59]. In addition to further elucidating the regulatory function of the N-terminal tails of PIF1 family helicases, other puzzling observations still await explanations. What is so special about genome maintenance in some yeasts that it requires two PIF1 helicases when eukaryotes with more complex genomes, including humans, cope with one? What extra functions make *S. pombe* Pfh1 essential for survival? Are these functions performed by other proteins in other eukaryotes or are these functions not required? Besides providing detailed insights into the growing number of cellular functions and biochemical characteristics of the PIF1 helicase family, yeast can also serve as a powerful model system for the functional evaluation of hPif1, and potentially disease-associated hPif1 mutations.

## Figures and Tables

**Table 1:** Yeast PIF1 helicase function in the maintenance of genomic integrity. Rrm3 and ScPif1 localize mainly to the same genomic sites in the yeast genome and have several overlapping functions. Green boxes indicate the ability to perform the function, red boxes mark the absence of the function, and yellow indicates a backup role. Pfh1 localizes to similar sites as ScPif1 and Rrm3, but several functions and associations with genomic loci known for Rrm3 and ScPif1 have not been tested for Pfh1. *Pif1 was not found to affect replication through telomeres when assayed by 2D gels [27], but the more recently discovered role of Pif1 in G4 unwinding might suggest such a role. Numbers indicate references to relevant studies. N.D. indicates not determined.

		*S. cerevesiae*			*S. pombe*
		Rrm3	Pif1		Pfh1
	**Essential**	No	No		Yes
	**Nucleus**	[28]	[28]		[28]
	**Mitochondria**	[29]	[28]		[28]
**Localization**	Telomeres	[27]	[30,31]		[32]
	mtDNA	N.D.	[33]		[28]
	rDNA	[16]	[16]		[16]
	tRNA genes	[34,35]	[35,36]		[17]
	Centromeres	[34,37]	[37]		N.D.
	Highly transcribed genes	[38]	[39]		[40,41]
	Active/Inactive DNA replication forks	[10]	[10]		N.D.
	Transcription-replication conflicts	[38]	[38]		[17]
	Rad52 DNA-damage foci	[28]	[28]		[28]
	Origins of replication	[16,42]	[16]		N.D.
**Function**	Replication through telomeres	[27]	N.D.*		[32]
	Telomere anchoring	[43]	[44]		N.D.
	Telomerase inhibition	[27]	[13]		[32]
	G4 structures	[45]	[39,46]		[41,47]
	Okazaki fragment maturation	[36]	[48]		[49]
	Centromere Replication and Segregation	[37]	[37]		N.D.
	Sister chromatid cohesion	[50]	[37]		N.D.
	Sister chromatid exchange	[51]	[51]		N.D.
	Break-induced replication	[11,52]	[11,52]		N.D.
	DNA synthesis restriction (HU)	[42]	N.D.		N.D.
	Fork convergence	[16,53]	[53]		[17,54]
	Daughter-strand gap repair	[51]	[55]		N.D.
	Silent mating type locus	[34]	N.D.		[17]
	Fork progression through tRNA genes	[35,36]	[35,36]		[17]
	Fork progression through rDNA	[16]	[16]		[17]
	Repression of Ty1 mobility	[56,57]	N.D.		N.D.
	Maintenance of mtDNA	[36]	[33,36]		[28]
	H2AX/H2A phosphorylation	N.D.	N.D.		[17]
